# *catena*-Poly[{μ-cyanido-bis­[(4,4′-dimethyl-2,2′-bipyridine-κ^2^ *N*,*N*′)copper(I)]}-μ-cyanido-copper(I)-μ-cyanido]

**DOI:** 10.1107/S1600536808022964

**Published:** 2008-07-26

**Authors:** Shi-Hong Lin, Ying-Yi Yang, Seik Weng Ng

**Affiliations:** a Department of Chemistry, Shantou University, Shantou, Guangdong 515063, People’s Republic of China; b Department of Chemistry, University of Malaya, 50603 Kuala Lumpur, Malaysia

## Abstract

In the title compound, [Cu_3_(CN)_3_(C_12_H_12_N_2_)_2_], two 2,2′-bipyridine *N*,*N*′-chelated Cu^I^ atoms are linked by a cyanide bridge that lies about a center of inversion; the Cu^I^ atom exists in a tetra­hedral coordination geometry. This dinuclear entity is linked to another Cu^I^ atom that lies on a twofold rotation axis by another cyanide bridge, these bridges giving rise to the formation of a linear chain motif.

## Related literature

Some copper(I) cyanide adducts with 2,2′-bipyridine-like ligands that adopt chain structures in which the cyanide group functions as a bridge are tris­cyano-bis­(2,2′-biquinoline)tri­cop­per (Chesnut *et al.*, 2001 ▶; Dessy *et al.*, 1985 ▶), tetra­kiscyano­(2,2′-biquinoline)tetra­copper (Chesnut & Zubieta, 1998 ▶) and bis­cyano-(4,4′-diphenyl-2,2′-bipyridine)dicopper (Chesnut *et al.*, 2001 ▶).
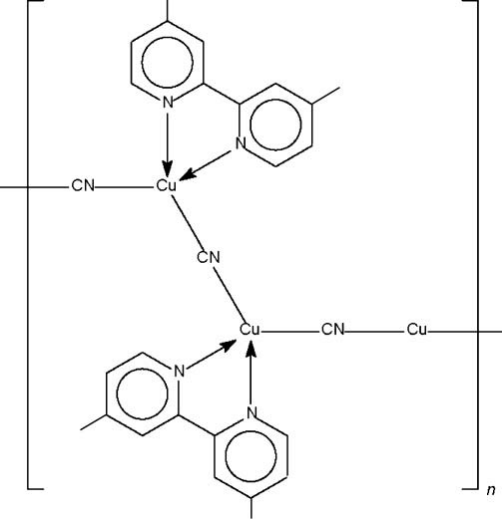


## Experimental

### 

#### Crystal data

[Cu_3_(CN)_3_(C_12_H_12_N_2_)_2_]
*M* *_r_* = 637.15
Monoclinic, 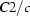

*a* = 10.7196 (7) Å
*b* = 12.3700 (9) Å
*c* = 20.9182 (14) Å
β = 100.146 (1)°
*V* = 2730.4 (3) Å^3^
*Z* = 4
Mo *K*α radiation
μ = 2.34 mm^−1^
*T* = 295 (2) K
0.30 × 0.20 × 0.16 mm

#### Data collection

Bruker SMART APEX diffractometer
Absorption correction: multi-scan (*SADABS*; Sheldrick, 1996 ▶) *T*_min_ = 0.540, *T*_max_ = 0.705
8685 measured reflections
3125 independent reflections
2491 reflections with *I* > 2σ(*I*)
*R*_int_ = 0.024

#### Refinement

*R*[*F* ^2^ > 2σ(*F* ^2^)] = 0.038
*wR*(*F* ^2^) = 0.110
*S* = 1.03
3125 reflections
170 parameters
H-atom parameters constrained
Δρ_max_ = 0.53 e Å^−3^
Δρ_min_ = −0.21 e Å^−3^

### 

Data collection: *SMART* (Bruker, 2002 ▶); cell refinement: *SAINT* (Bruker, 2002 ▶); data reduction: *SAINT*; program(s) used to solve structure: *SHELXS97* (Sheldrick, 2008 ▶); program(s) used to refine structure: *SHELXL97* (Sheldrick, 2008 ▶); molecular graphics: *X-SEED* (Barbour, 2001 ▶); software used to prepare material for publication: *publCIF* (Westrip, 2008 ▶).

## Supplementary Material

Crystal structure: contains datablocks global, I. DOI: 10.1107/S1600536808022964/rk2103sup1.cif

Structure factors: contains datablocks I. DOI: 10.1107/S1600536808022964/rk2103Isup2.hkl

Additional supplementary materials:  crystallographic information; 3D view; checkCIF report

## Figures and Tables

**Fig. 1.**
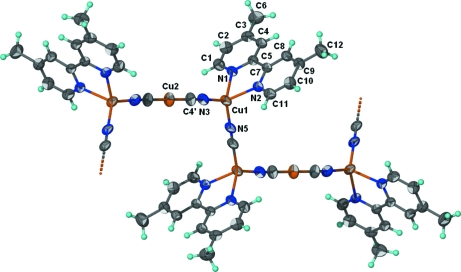
Thermal ellipsoid plot (Barbour, 2001) of a portion of the linear chain motif; probability levels are set at 50%.

## supplementary crystallographic information

### Comment

The cyanide group functions as a bridging group in a number of copper(I) adducts of 2,2'-bipyridine type of *N*-heterocycles. Those who crystal structure have been described include the triscyano-bis(2,2'-biquinoline)tricopper (Chesnut *et al.*, 2001; Dessy *et al.*, 1985), tetrakiscyano(2,2'-biquinoline)tetracopper (Chesnut & Zubieta, 1998) and biscyano-(4,4'-diphenyl-2,2'-bipyridine)dicopper (Chesnut *et al.*, 2001).

The copper(I) cyanide adduct with 4,4'-dimethyl-2,2'-biyridine (Scheme 1, Fig. 1) adopts a similar chain motif. Two *N*-heterocycle-chelated copper(I) atoms are linked by a cyanide bridge that lies about a center-of-inversion; the copper(I) atom exists in a tetrahedral coordination geometry. This dinuclear entity is linked to copper(I) atom that lies on a twofold rotation axis by another cyanide bridge.

### Experimental

4,4'-Dimethyl-2,2'-bipyridine (0.055 g, 0.3 mmol), cuprous cyanide (0.009 g, 0.1 mmol) and acetonitrile (8 ml) were placed in a 15-ml, teflon-lined autoclave. It was heated at 453 K for 72 hours, then was cooled to 333 K at a rate of 5 K per hour and then kept at this temperature for a further 10 hours before being cooled to room temperature. Red prisms were in 50% yield based on the *N*-heterocycle.

### Refinement

The component atoms of the cyanide groups were each refined as a 50%:50% mixture of carbon and nitrogen. The pair of C/N atoms were restrained to the same site and also to have the same temperature factors. Hydrogen atoms were placed at calculated positions in the riding model approximation with C—H = 0.93–0.98 Å and U_iso_(H) = 1.2–1.5U_eq_(C).

### Crystal data

**Table:**

[Cu_3_(CN)_3_(C_12_H_12_N_2_)_2_]	*F*_000_ = 1288
*M**_r_* = 637.15	*D*_x_ = 1.550 Mg m^−^^3^
Monoclinic, *C*2/*c*	Mo *K*α radiation λ = 0.71073 Å
Hall symbol: -C 2yc	Cell parameters from 2750 reflections
*a* = 10.7196 (7) Å	θ = 2.5–27.0º
*b* = 12.3700 (9) Å	µ = 2.34 mm^−^^1^
*c* = 20.9182 (14) Å	*T* = 295 (2) K
β = 100.146 (1)º	Block, red
*V* = 2730.4 (3) Å^3^	0.30 × 0.20 × 0.16 mm
*Z* = 4	

### Data collection

**Table:**

Bruker SMART APEX diffractometer	3125 independent reflections
Radiation source: fine-focus sealed tube	2491 reflections with *I* > 2σ(*I*)
Monochromator: graphite	*R*_int_ = 0.024
*T* = 295(2) K	θ_max_ = 27.5º
φ and ω scans	θ_min_ = 2.5º
Absorption correction: multi-scan(SADABS; Sheldrick, 1996)	*h* = −13→13
*T*_min_ = 0.540, *T*_max_ = 0.705	*k* = −16→15
8685 measured reflections	*l* = −16→27

### Refinement

**Table:**

Refinement on *F*^2^	Secondary atom site location: difference Fourier map
Least-squares matrix: full	Hydrogen site location: inferred from neighbouring sites
*R*[*F*^2^ > 2σ(*F*^2^)] = 0.038	H-atom parameters constrained
*wR*(*F*^2^) = 0.110	*w* = 1/[σ^2^(*F*_o_^2^) + (0.065*P*)^2^ + 0.8323*P*] where *P* = (*F*_o_^2^ + 2*F*_c_^2^)/3
*S* = 1.03	(Δ/σ)_max_ = 0.001
3125 reflections	Δρ_max_ = 0.53 e Å^−^^3^
170 parameters	Δρ_min_ = −0.21 e Å^−^^3^
Primary atom site location: structure-invariant direct methods	Extinction correction: none

### Fractional atomic coordinates and isotropic or equivalent isotropic displacement parameters (Å^2^)

**Table:**

	*x*	*y*	*z*	*U*_iso_*/*U*_eq_	Occ. (<1)
Cu1	0.74268 (3)	0.41078 (2)	0.072850 (16)	0.04716 (14)	
Cu2	0.5000	0.41827 (4)	0.2500	0.05469 (16)	
N1	0.75592 (19)	0.54384 (16)	0.01065 (10)	0.0420 (5)	
N2	0.92273 (18)	0.47469 (15)	0.11124 (9)	0.0406 (4)	
N3	0.6442 (3)	0.41773 (18)	0.14085 (14)	0.0519 (6)	0.50
N4	0.5915 (2)	0.4184 (2)	0.18400 (12)	0.0502 (6)	0.50
N5	0.7489 (2)	0.28632 (17)	0.01721 (11)	0.0487 (6)	0.50
C3'	0.6442 (3)	0.41773 (18)	0.14085 (14)	0.0519 (6)	0.50
C4'	0.5915 (2)	0.4184 (2)	0.18400 (12)	0.0502 (6)	0.50
C5'	0.7489 (2)	0.28632 (17)	0.01721 (11)	0.0487 (6)	0.50
C1	0.6717 (3)	0.5725 (2)	−0.04135 (14)	0.0531 (6)	
H1	0.5932	0.5384	−0.0485	0.064*	
C2	0.6951 (3)	0.6501 (2)	−0.08494 (13)	0.0562 (7)	
H2	0.6325	0.6680	−0.1200	0.067*	
C3	0.8105 (3)	0.7011 (2)	−0.07670 (12)	0.0493 (6)	
C4	0.8992 (2)	0.67121 (18)	−0.02273 (12)	0.0450 (6)	
H4	0.9791	0.7030	−0.0154	0.054*	
C5	0.8687 (2)	0.59363 (16)	0.02044 (12)	0.0385 (5)	
C6	0.8409 (4)	0.7858 (2)	−0.12307 (15)	0.0699 (9)	
H6A	0.8117	0.7623	−0.1669	0.105*	
H6B	0.9309	0.7970	−0.1164	0.105*	
H6C	0.7997	0.8523	−0.1156	0.105*	
C7	0.9594 (2)	0.56084 (18)	0.07973 (11)	0.0391 (5)	
C8	1.0699 (2)	0.61388 (19)	0.10154 (13)	0.0461 (6)	
H8	1.0910	0.6739	0.0789	0.055*	
C9	1.1516 (3)	0.5801 (2)	0.15691 (14)	0.0523 (6)	
C10	1.1131 (3)	0.4918 (2)	0.18924 (14)	0.0559 (7)	
H10	1.1634	0.4661	0.2270	0.067*	
C11	1.0006 (3)	0.4430 (2)	0.16498 (13)	0.0516 (6)	
H11	0.9766	0.3837	0.1873	0.062*	
C12	1.2752 (3)	0.6366 (3)	0.18039 (18)	0.0781 (10)	
H12A	1.3038	0.6203	0.2255	0.117*	
H12B	1.2635	0.7133	0.1751	0.117*	
H12C	1.3372	0.6125	0.1556	0.117*	

### Atomic displacement parameters (Å^2^)

**Table:**

	*U*^11^	*U*^22^	*U*^33^	*U*^12^	*U*^13^	*U*^23^
Cu1	0.0535 (2)	0.0446 (2)	0.0483 (2)	−0.01261 (12)	0.02281 (16)	−0.00397 (12)
Cu2	0.0494 (3)	0.0793 (4)	0.0406 (3)	0.000	0.0223 (2)	0.000
N1	0.0483 (11)	0.0401 (10)	0.0402 (12)	−0.0070 (8)	0.0149 (9)	−0.0055 (8)
N2	0.0469 (10)	0.0416 (10)	0.0376 (11)	−0.0031 (8)	0.0193 (9)	0.0007 (8)
N3	0.0530 (13)	0.0527 (14)	0.0522 (15)	−0.0098 (10)	0.0152 (12)	−0.0068 (10)
N4	0.0469 (13)	0.0700 (16)	0.0387 (13)	−0.0049 (10)	0.0214 (11)	−0.0049 (10)
N5	0.0560 (13)	0.0453 (12)	0.0514 (15)	−0.0154 (10)	0.0281 (11)	−0.0066 (9)
C3'	0.0530 (13)	0.0527 (14)	0.0522 (15)	−0.0098 (10)	0.0152 (12)	−0.0068 (10)
C4'	0.0469 (13)	0.0700 (16)	0.0387 (13)	−0.0049 (10)	0.0214 (11)	−0.0049 (10)
C5'	0.0560 (13)	0.0453 (12)	0.0514 (15)	−0.0154 (10)	0.0281 (11)	−0.0066 (9)
C1	0.0539 (15)	0.0559 (15)	0.0491 (16)	−0.0090 (12)	0.0082 (13)	−0.0071 (12)
C2	0.0655 (16)	0.0599 (16)	0.0415 (15)	0.0020 (13)	0.0047 (13)	0.0003 (12)
C3	0.0678 (16)	0.0432 (12)	0.0409 (14)	0.0022 (12)	0.0206 (13)	0.0002 (10)
C4	0.0525 (14)	0.0423 (13)	0.0451 (14)	−0.0055 (10)	0.0221 (11)	−0.0005 (10)
C5	0.0457 (12)	0.0370 (11)	0.0365 (13)	−0.0020 (9)	0.0175 (10)	−0.0035 (9)
C6	0.098 (2)	0.0630 (18)	0.0550 (19)	0.0058 (16)	0.0312 (17)	0.0142 (14)
C7	0.0456 (13)	0.0367 (11)	0.0400 (13)	−0.0031 (9)	0.0216 (11)	−0.0021 (9)
C8	0.0501 (14)	0.0424 (12)	0.0482 (15)	−0.0047 (10)	0.0153 (12)	0.0046 (10)
C9	0.0506 (14)	0.0537 (15)	0.0530 (17)	−0.0049 (11)	0.0103 (12)	−0.0013 (11)
C10	0.0554 (15)	0.0640 (17)	0.0473 (16)	0.0013 (13)	0.0066 (12)	0.0101 (13)
C11	0.0628 (16)	0.0484 (13)	0.0474 (15)	−0.0050 (12)	0.0201 (13)	0.0069 (12)
C12	0.0605 (18)	0.080 (2)	0.086 (2)	−0.0172 (16)	−0.0074 (17)	0.0093 (19)

### Geometric parameters (Å, °)

**Table:**

Cu1—N1	2.118 (2)	C4—C5	1.395 (3)
Cu1—N2	2.109 (2)	C4—H4	0.9300
Cu1—N3	1.917 (3)	C5—C7	1.491 (3)
Cu1—N5	1.938 (2)	C6—H6A	0.9600
Cu2—N4	1.829 (2)	C6—H6B	0.9600
Cu2—N4^i^	1.829 (2)	C6—H6C	0.9600
N1—C1	1.333 (3)	C7—C8	1.361 (4)
N1—C5	1.340 (3)	C8—C9	1.388 (4)
N2—C11	1.336 (3)	C8—H8	0.9300
N2—C7	1.347 (3)	C9—C10	1.385 (4)
N3—N4	1.146 (4)	C9—C12	1.502 (4)
N5—C5'^ii^	1.154 (4)	C10—C11	1.364 (4)
C1—C2	1.377 (4)	C10—H10	0.9300
C1—H1	0.9300	C11—H11	0.9300
C2—C3	1.372 (4)	C12—H12A	0.9600
C2—H2	0.9300	C12—H12B	0.9600
C3—C4	1.392 (4)	C12—H12C	0.9600
C3—C6	1.503 (4)		
			
N3—Cu1—N5	124.25 (9)	N1—C5—C4	121.7 (2)
N3—Cu1—N2	106.70 (9)	N1—C5—C7	116.1 (2)
N5—Cu1—N2	113.61 (9)	C4—C5—C7	122.2 (2)
N3—Cu1—N1	121.75 (9)	C3—C6—H6A	109.5
N5—Cu1—N1	103.61 (9)	C3—C6—H6B	109.5
N2—Cu1—N1	77.69 (7)	H6A—C6—H6B	109.5
N4—Cu2—C4'^i^	179.88 (15)	C3—C6—H6C	109.5
C4'^i^—Cu2—N4^i^	0.00 (12)	H6A—C6—H6C	109.5
C1—N1—C5	117.7 (2)	H6B—C6—H6C	109.5
C1—N1—Cu1	126.79 (17)	N2—C7—C8	121.9 (2)
C5—N1—Cu1	114.80 (16)	N2—C7—C5	114.8 (2)
C11—N2—C7	116.8 (2)	C8—C7—C5	123.3 (2)
C11—N2—Cu1	127.21 (16)	C7—C8—C9	121.3 (2)
C7—N2—Cu1	115.86 (16)	C7—C8—H8	119.4
N4—N3—Cu1	175.6 (3)	C9—C8—H8	119.4
N3—N4—Cu2	177.1 (3)	C10—C9—C8	116.5 (2)
C5'^ii^—N5—Cu1	178.3 (3)	C10—C9—C12	121.9 (3)
N5^ii^—N5—Cu1	178.3 (3)	C8—C9—C12	121.6 (3)
N1—C1—C2	123.4 (2)	C11—C10—C9	119.2 (3)
N1—C1—H1	118.3	C11—C10—H10	120.4
C2—C1—H1	118.3	C9—C10—H10	120.4
C3—C2—C1	120.2 (3)	N2—C11—C10	124.3 (2)
C3—C2—H2	119.9	N2—C11—H11	117.8
C1—C2—H2	119.9	C10—C11—H11	117.8
C2—C3—C4	116.8 (2)	C9—C12—H12A	109.5
C2—C3—C6	122.2 (3)	C9—C12—H12B	109.5
C4—C3—C6	120.9 (2)	H12A—C12—H12B	109.5
C3—C4—C5	120.2 (2)	C9—C12—H12C	109.5
C3—C4—H4	119.9	H12A—C12—H12C	109.5
C5—C4—H4	119.9	H12B—C12—H12C	109.5
			
N3—Cu1—N1—C1	−81.2 (2)	C1—N1—C5—C7	178.9 (2)
N5—Cu1—N1—C1	64.9 (2)	Cu1—N1—C5—C7	−9.9 (2)
N2—Cu1—N1—C1	176.6 (2)	C3—C4—C5—N1	1.8 (3)
N3—Cu1—N1—C5	108.54 (18)	C3—C4—C5—C7	−178.5 (2)
N5—Cu1—N1—C5	−105.30 (17)	C11—N2—C7—C8	−0.4 (3)
N2—Cu1—N1—C5	6.36 (15)	Cu1—N2—C7—C8	176.04 (18)
N3—Cu1—N2—C11	54.7 (2)	C11—N2—C7—C5	−179.5 (2)
N5—Cu1—N2—C11	−85.9 (2)	Cu1—N2—C7—C5	−3.0 (2)
N1—Cu1—N2—C11	174.5 (2)	N1—C5—C7—N2	8.7 (3)
N3—Cu1—N2—C7	−121.34 (17)	C4—C5—C7—N2	−171.0 (2)
N5—Cu1—N2—C7	98.09 (17)	N1—C5—C7—C8	−170.4 (2)
N1—Cu1—N2—C7	−1.55 (15)	C4—C5—C7—C8	10.0 (4)
C5—N1—C1—C2	0.0 (4)	N2—C7—C8—C9	1.3 (4)
Cu1—N1—C1—C2	−170.0 (2)	C5—C7—C8—C9	−179.7 (2)
N1—C1—C2—C3	1.1 (4)	C7—C8—C9—C10	−1.5 (4)
C1—C2—C3—C4	−0.7 (4)	C7—C8—C9—C12	178.5 (3)
C1—C2—C3—C6	179.7 (3)	C8—C9—C10—C11	1.0 (4)
C2—C3—C4—C5	−0.7 (4)	C12—C9—C10—C11	−179.1 (3)
C6—C3—C4—C5	179.0 (2)	C7—N2—C11—C10	−0.2 (4)
C1—N1—C5—C4	−1.4 (3)	Cu1—N2—C11—C10	−176.2 (2)
Cu1—N1—C5—C4	169.76 (17)	C9—C10—C11—N2	−0.1 (5)

Symmetry codes: (i) −*x*+1, *y*, −*z*+1/2; (ii) −*x*+3/2, −*y*+1/2, −*z*.
